# Rating knockdown of flour beetles after exposure to two insecticides as an indicator of mortality

**DOI:** 10.1038/s41598-020-78982-z

**Published:** 2021-01-13

**Authors:** Christos G. Athanassiou, Nickolas G. Kavallieratos, Frank H. Arthur, Christos T. Nakas

**Affiliations:** 1 Laboratory of Entomology and Agricultural Zoology, Department of Agriculture, Crop Production and Rural Environment, University of Thessaly, Phytokou Str., 38446 Nea Ionia, Magnesia, Greece; 2 Center for Grain and Animal Health Research, United States Department of Agriculture, Agricultural Research Service, 1515 College Avenue, Manhattan, KS 66502 USA; 3 Laboratory of Agricultural Zoology and Entomology, Department of Crop Science, Agricultural University of Athens, 75 Iera Odos Str., 11855 Athens, Greece; 4 Laboratory of Biometry, Department of Agriculture, Crop Production and Rural Environment, University of Thessaly, Phytokou Str., 38446 Nea Ionia, Magnesia, Greece; 5 University Institute of Clinical Chemistry, Inselspital, Bern University Hospital, University of Bern, Bern, Switzerland; 6 Retired, Manhattan, Kansas USA

**Keywords:** Entomology, Biological techniques

## Abstract

Knockdown and mortality of adults of the red flour beetle, *Tribolium castaneum* (Herbst) and the confused flour beetle, *Tribolium confusum* Jacquelin du Val, were assessed after exposure to two contact insecticides, chlorfenapyr and cyfluthrin, on a concrete surface. Individuals were rated on a scale for knockdown of exposed adults according to their mobility from 1, representing immobilized adults to 5, representing normally moving (similar to the controls). Only cyfluthrin gave immediate knockdown. Adults were rated at 1, 3 and 7 days post-exposure. After the final assessment, adults were discarded and the same procedure was repeated for 5 consecutive weeks with new adults exposed on the same treated surfaces. Despite initial knockdown, many individuals did not eventually die after exposure to cyfluthrin. In contrast, adults exposed to chlorfenapyr were not initially knocked down after exposure but most died after 7 days. These trends were similar during the entire 5-week residual testing period. The storage of the treated dishes in illuminated or non-illuminated conditions did not affect the insecticidal effect of either insecticide. The results of the present study can be further implemented towards the design of a “lethality index” that can serve as a quick indicator of knockdown and mortality rates caused after exposure to insecticides.

## Introduction

Efficacy of insecticides is often based on the assessment of insect mortality, which is a critical factor in testing the effectiveness of insecticides in both laboratory and field tests. However, there are some insecticides that do not cause direct mortality to exposed adults, such as insect growth regulators (IGRs), but instead affect molting and development of immature stages^1,2^. Most contact insecticides act on the nervous system of insects and can alter behavior before death. For most contact insecticides, the most important behavioral change after an adult insect is exposed is “knockdown”, which is defined as paralysis, whether reversible or not^3–6^. In an earlier study, Leskey et al.^7^ evaluated 37 insecticidal treatments for the control of the brown marmorated stink bug, *Halyomorpha halys* (Stal) (Hemiptera: Pentatomidae) and found that increased knockdown of the exposed individuals was directly related to increased insecticidal efficacy, which in turn was related to increasing exposure interval. However, in another study, Zhu et al.^8^ emphasized the widespread development of “knockdown resistance” (kdr) to pyrethroids, using the bed bug, *Cimex lectularius* L. (Hemiptera: Cimicidae), as an example, through a sodium channel mutation. Similar results have been reported by Haddi et al.^9^ for kdr patterns of stored product beetles.

In pest management programs for stored products, there are several contact insecticides that can be used to manage adult insects. Most of the published studies regarding the efficacy of these insecticides are focused solely on the mortality of the target individuals after exposure on a treated substrate^6, 10–12^. For short-term exposures of adults of the lesser grain borer, *Rhyzopertha dominica* (F.) (Coleoptera: Bostrychidae) on wheat treated with a mixture of chlorpyriphos-methyl with deltamethrin, Arthur^13^ noted increased survival after the removal of the treated commodity. Similarly, adults of the rice weevil, *Sitophilus oryzae* (L.) (Coleoptera: Curculionidae) that had been removed from spinosad-treated commodities were able to survive and continue to cause grain damage^14^. However, when Tsaganou et al.^15^ tested the neonicotinoid thiamethoxam for the control of five stored-product beetle species, they found that knockdown was extremely low for four of them, but it was high for the larger grain borer, *Prostephanus truncatus* (Horn) (Coleoptera: Bostrychidae). Most individuals of this species that had been exposed for periods shorter than 72 h were able to survive. Hence, the relationship between knockdown and mortality is species-specific.

While knockdown is generally considered an indicator that insects respond to a specific insecticide, it may be also related to reversible enzyme inhibition or detoxication mechanisms. Consequently, it is questionable whether and how knockdown contributes to insecticidal efficacy. Theoretically, knockdown causes the interruption of the contact of the insect with the lethal agent through reduced motor activity^3,16^. In this context, quick knockdown, as in the case of pyrethroids, may increase the possibility for recovery. Conversely, quick knockdown minimizes the chances that the exposed insect has to move away from the treated substrate and recover. In this context, Arthur^13^ noted that recovery of insects after exposure is very likely to appear in most of the typical neurotoxic insecticides. However, in most of the studies available that assess knockdown, especially in the case of stored-product insect species, knockdown is recorded as an intermediate stage between life and death^13,17^. Knockdown has no standard scaling, and can vary remarkably between irregular walking, where the insect is able to walk but with interruptions, and inability to walk, where the insect shows only a minimal movement, i.e. a slight movement of the antennae or the tarsi. When adult insects are exposed to neurotoxic insecticides, it is not clear whether a state of knockdown that allows crawling will eventually result in increased levels of recovery, or, conversely, if a state of knockdown that allows only a minimal movement will result in increased mortality. Consequently, knockdown can be a dynamic condition that changes over time and has a certain plasticity between “mobility” and “immobility”, but it not clear if and how knockdown relates to mortality or recovery as a final outcome. Arthur^13^ noted that adults of the red flour beetle, *Tribolium castaneum* (Herbst) (Coleoptera: Tenebrionidae) that had access to food, were able to recover despite the fact that they were knocked down. In addition, Athanassiou et al.^18^ for a combination of beta-cyfluthrin with imidacloprid that had been applied on concrete, noted that although knockdown of *T. castaneum* and the confused flour beetle, *Tribolium confusum* Jacquelin du Val (Coleoptera: Tenebrionidae) was rapid, mortality after 7 d of exposure was generally low. This indicates that the rapidity of knockdown does not always correspond to the rapidity of mortality.

The residual efficacy of insecticides applied to flooring surfaces in milling and processing facilities can decline with time, due to the influence of several biotic or abiotic factors, including susceptibility of the target species, initial concentration of insecticide, or time post-application. Light intensity inside a facility may also affect residual efficacy. These changes may also alter the ratio or the levels of knockdown, as increase of walking ability after the initial immobilization may suggest that the outcome of knockdown is reversible. Guedes et al.^17^ observed the movement of two stored-product psocid species, *Liposcelis bostrychophila* Badonnel (Psocoptera: Liposcelididae) and *Liposcelis entomophila* (Enderlein) (Psocoptera: Liposcelididae) after exposure to several insecticides. In that study, the authors found that movement after exposure varied remarkably among insecticides for both species. Mobility was reduced on a surface that had been treated with pyrethroids, despite the fact that it is generally expected that the neurotoxic activity of these insecticides increases mobility^6,17^.

The objective of this study was to assess the different states of knockdown, and their relationship with the efficacy of insecticides on a concrete surface. For this purpose, we selected two insecticides, the pyrethroid beta-cyfluthrin and the pyrrole chlorfenapyr. We also used two stored-product beetles as our model insects, *T. confusum* and *T. castaneum*, which are common pests of milling and processing facilities. Factors that affect knockdown and mortality, including the concentration of insecticide, the exposure interval, and the residual time post-application were included in the study. As there is limited data in the scientific literature on effects of photoperiod on knockdown and mortality, this factor was also included in the study.

## Results

### Knockdown

**Figure 1:**
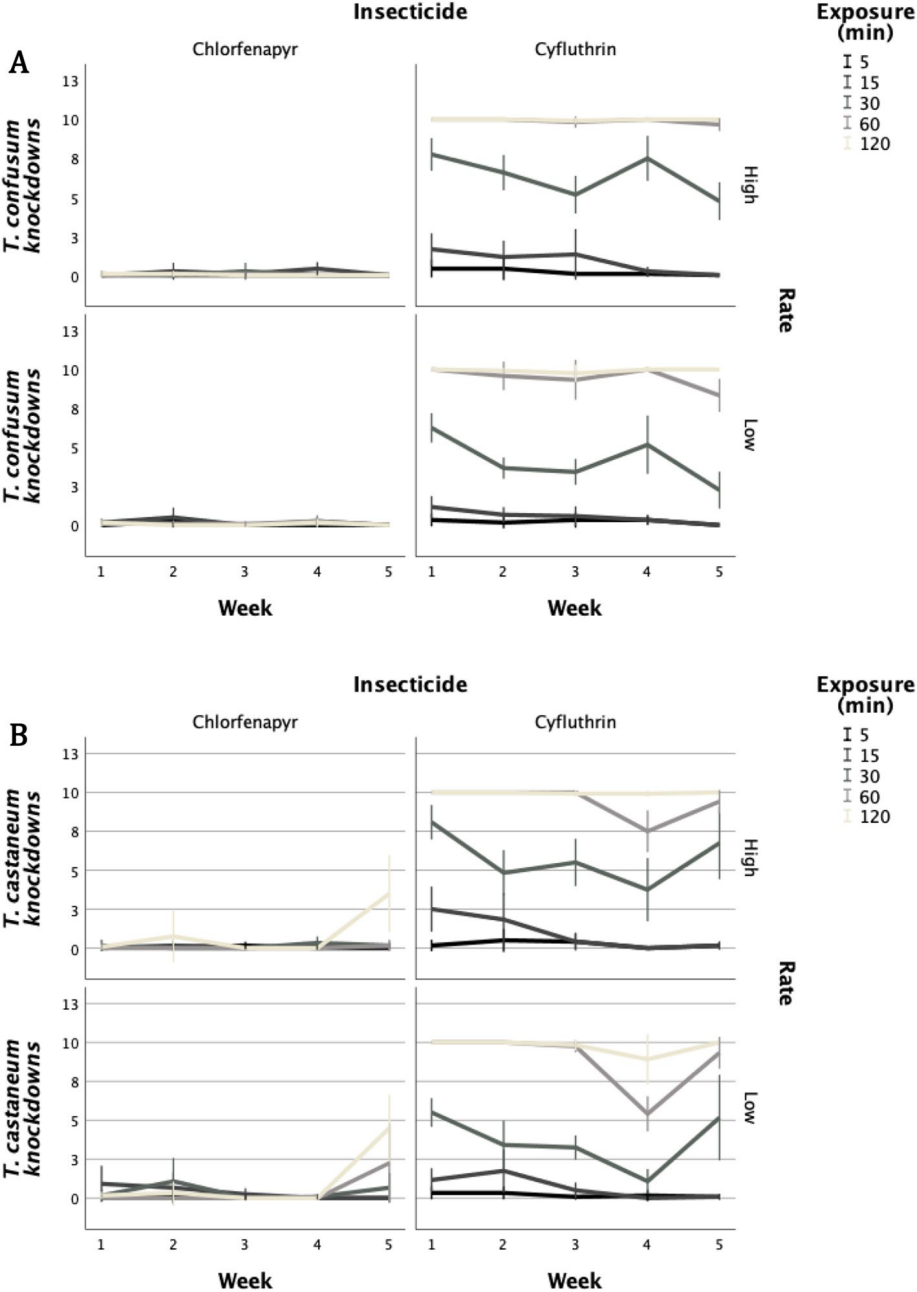
Knockdown of each species exposed on two insecticides, applied at two rates, at different exposure intervals (y-axis indicates mean number of adults out of 10, (**a**) *T. confusum*, (**b**) *T. castaneum*). Knockdown is expressed as number of individuals out of the 10 that had been exposed.

Knockdown patterns immediately after exposure were different for *T. castaneum* and *T. confusum* (Table 1). Light was the only factor that did not affect knockdown for *T. confusum* exposed to cyfluthrin, while the only factor affecting *T. castaneum* knockdown after exposure to cyfluthrin was the exposure time. There was no knockdown of either species at any time after exposure to chlorfenapyr (Fig. 1). Knockdown after exposure to cyfluthrin occurred after the shortest exposure time of 15 min, and generally exceeded 60% after 60 min. Nearly all adults were immobilized at the end of the observation period (120 min). This trend remained for all weeks examined, but during the last week, time to knockdown was longer. Knockdown of *T. confusum* adults was generally faster in comparison with adults of *T. castaneum*.

**Table 1:** Significance of effects for knockdown rates of the two species in a multivariable approach.

Source (*df*)	*T. castaneum*		*T. confusum*	
	χ^2^	*p*	χ^2^	*p*
Week (1)	7.0	0.008	1.5	0.221
Light (2)	0.9	0.638	2.7	0.259
Insecticide (1)	718.3	< 0.001	744.2	< 0.001
Rate (1)	4.4	0.036	2.0	0.157
Exposure (min) (1)	120.8	< 0.001	655.0	< 0.001

### Index values and mortality

**Figure 2:**
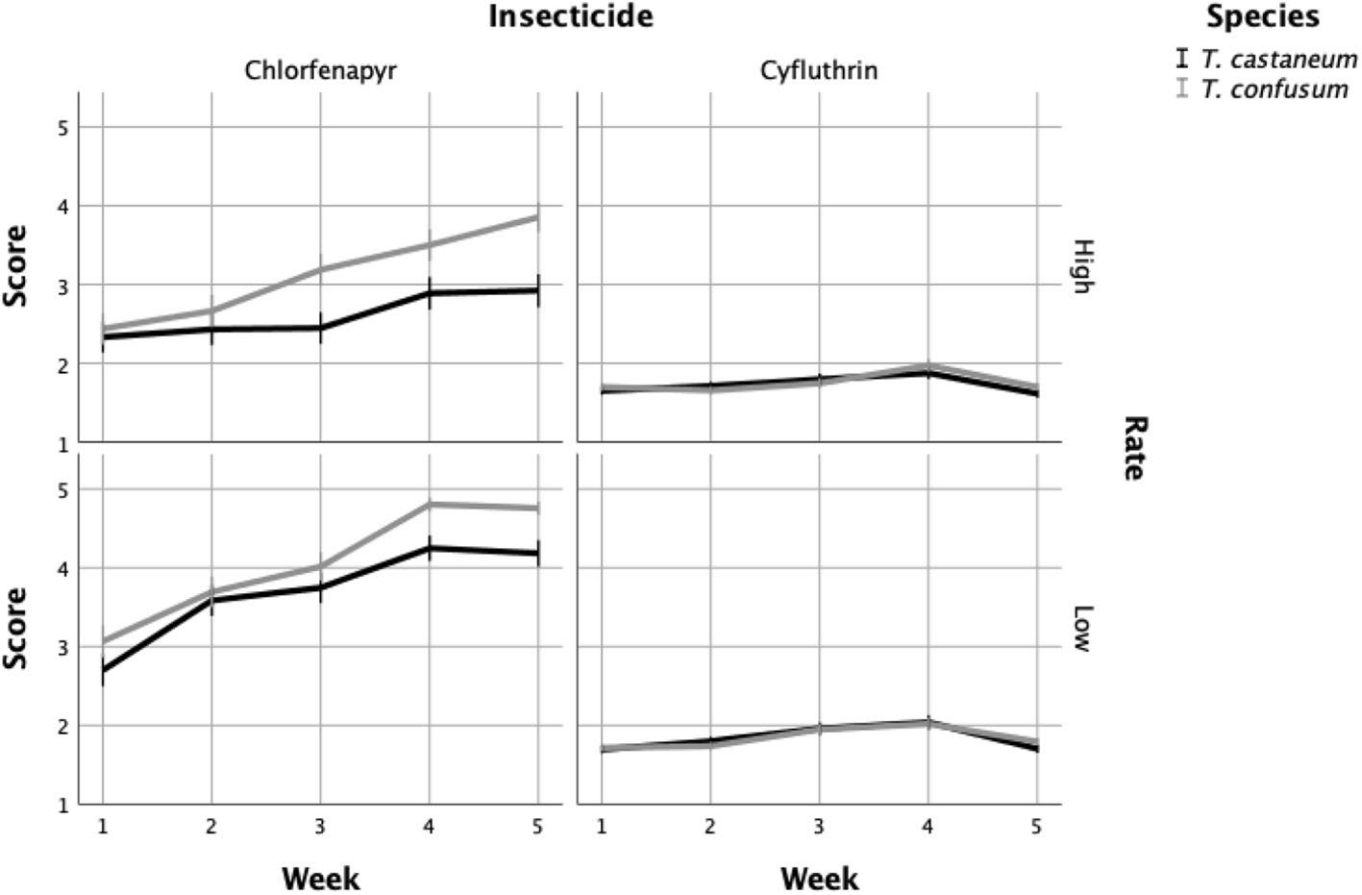
Mortality index of each species during the experimental period, for each insecticide and rate (1 = dead, 5 = normally moving).

For both species, insecticide and exposure time were highly significant (*p* < 0.001), while concentration was marginally significant for *T. castaneum* (*p* = 0.036) and non-significant for *T. confusum* (*p* = 0.161). All explanatory variables significantly affected the 1–5 scoring (*p* < 0.001 Table 2). However, when the analysis was run separately for each insecticide, light and species were not significant for cyfluthrin, but all factors were significant for chlorfenapyr (Table 3). Both insecticides showed similar trends during the entire observation period (Fig. 2). For chlorfenapyr, the index value was generally higher for *T. confusum* compared to *T. castaneum*, suggesting that *T. confusum* was the more susceptible species. Additionally, the difference between the two species, as shown by the index value, increased during the 5-week experimental period. Moreover, for both species, the index values for the low concentration of chlorfenapyr notably increased at the last weeks of observation, suggesting that the values were getting closer to “5”, indicating a loss of efficacy with time. Conversely, the index values for *T. confusum* exposed to the high chlorfenapyr concentration remained rather stable, with values close to 3, indicating little loss of efficacy with time. Similarly, for *T. castaneum*, the index value was generally close to 3 during the initial weeks of the period, with a slight decrease late in the observation period.

**Table 2:** ANOVA factors’ significance for main effects for both species together (total *df* = 14,253).

Source	*df*	F	*p*
Week	1	867.0	< 0.001
Light	2	20.6	< 0.001
Exposure	2	4466.1	< 0.001
Insecticide	1	7316.2	< 0.001
Rate	1	955.0	< 0.001
Species	1	157.7	< 0.001

**Table 3:** ANOVA factors’ significance for main effects for both species together, separately for each insecticide (for cyfluthrin total *df* = 7100, for chlorfenapyr total *df* = 7152).

Source	*df*	Cyfluthrin		Chlorfenapyr	
		F	*p*	F	*p*
Week	1	99.9	< 0.001	1017.7	< 0.001
Light	2	0.9	0.413	30.2	< 0.001
Exposure	2	4679.5	< 0.001	3748.1	< 0.001
Rate	1	131.3	< 0.001	1099.9	< 0.001
Species	1	1.6	0.202	225.3	< 0.001

The index values for cyfluthrin were notably lower in comparison with chlorfenapyr, and did not exceed 2 during the entire experimental period (Fig. 2). Both species had a “hard” knockdown during the experimental period and there was no recovery to “3” or higher. The high concentration generally decreased the index value, and thus resulted in increased mortality. However, the fact that knockdown index was not 1 suggests that a proportion of adults that were exposed was still alive after the 7-days exposure.

**Figure 3:**
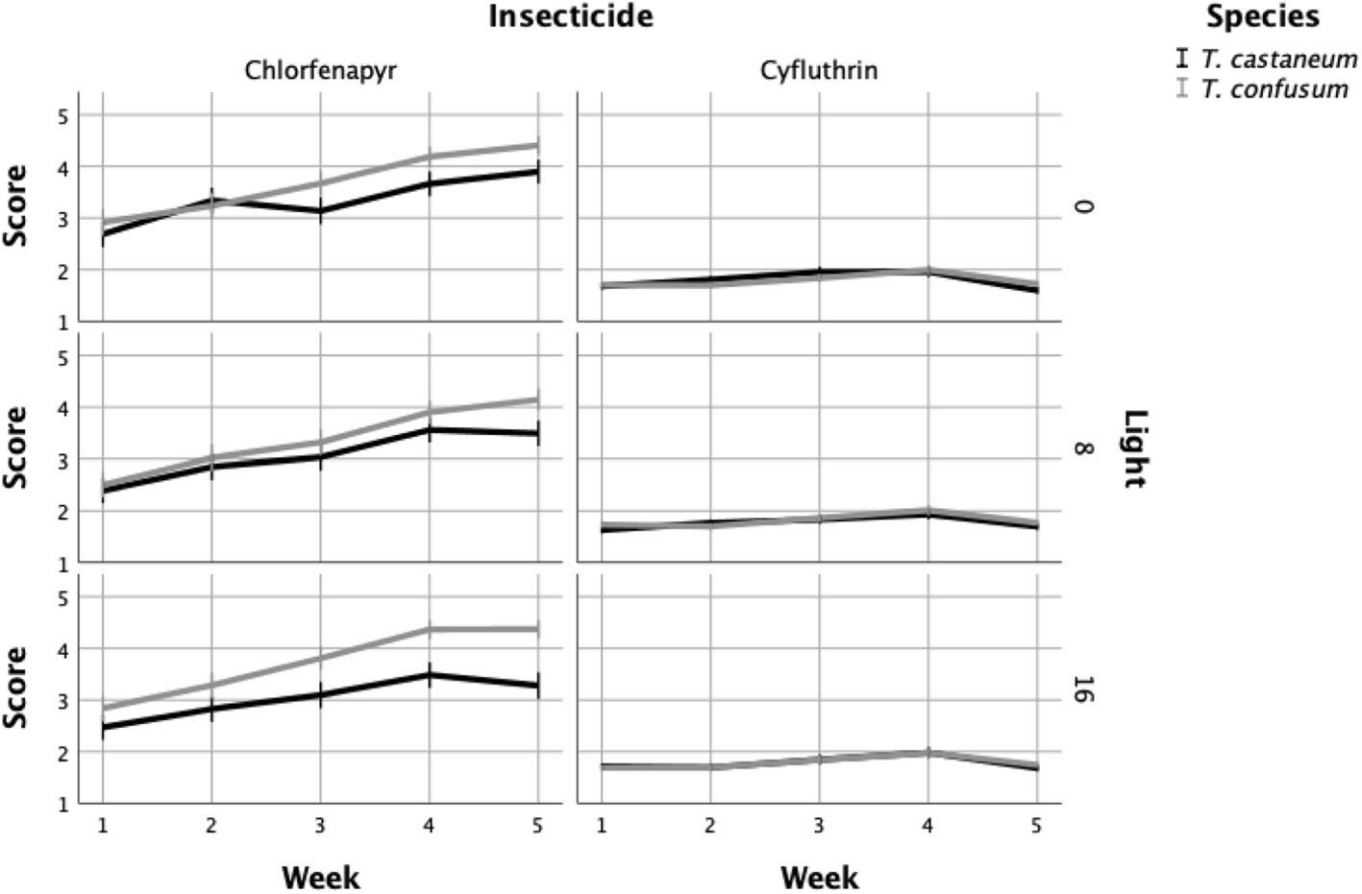
Mortality index of each species during the experimental period, for each insecticide and illumination condition (1 = dead, 5 = normally moving).

Similar patterns were also recorded when the data were analyzed for light condition (Fig. 3). For chlorfenapyr, *T. confusum* was less susceptible than *T. castaneum* at three conditions examined, but this difference was more apparent at 16:8. Moreover, the index values for cyfluthrin was rather linear, and close to 2 throughout the entire period. Generally, in contrast to results for chlorfenapyr, the index values for both species were similar for cyfluthrin. In general, the averaged index values for arenas that were held in continuous darkness were significantly lower than those for the other two light conditions. The light:dark conditions had more of an effect on chlorfenapyr than on cyfluthrin, but generally the differences were minor (mean index values: 2.68 for 0 h light, vs 2.61 and 2.54 for 16 and 8 h light, respectively).

## Discussion

In this study we compared two insecticides for residual efficacy, but also compared their effects on knockdown, and how knockdown can be used as an indicator of insect mortality. In general, rapid knockdown is considered an indicator of insecticide effectiveness. But, in recent studies with stored-product insects, knockdown was not related to mortality, because of the possibility for recovery. Arthur and Fontenot^11^ exposed immature *T. castaneum* in arenas treated with chlorfenapyr and found that survival was higher when adults had access to food, despite initial knockdown, and survival also increased if knockdown occurred in untreated areas of experimental arenas. Earlier, Arthur^19,20^ noted that even when knockdown of *T. castaneum* and *T. confusum* was high, recovery was also high during a post-exposure period, especially if adults were transferred to untreated arenas to assess recovery. However, for stored-grains, Athanassiou et al.^14^ noted delayed mortality of *R. dominica* adults after removal from a spinosad-treated substrate, indicating that even a short exposure was lethal. Conversely, with newer insecticides with novel modes of action such as chlorfenapyr, the factors that affect delayed mortality are poorly understood. For chlorfenapyr, our study shows that there was no knockdown, but mortality occurred after several days of continuous contact with the insecticide. The exposed adults of *T. castaneum* and *T. confusum* to chlorfenapyr were able to move normally, which means that there was no knockdown classified according to our index as 2, 3, or 4, and they progressed directly from normal movement (5) to dead (1). But, the absence of knockdown indicates that since the insects are able to normally move after exposure, they can escape from the treated area and colonize untreated areas. Still, the patterns of delayed mortality for these insecticides should be investigated more thoroughly, on the basis of examining the hypothesis if short exposures lead to delayed mortality.

Despite the fact that the index gives an estimation of the lethality through a classification of knockdown, the rationale of this index may be different if the values of this index, which are calculated per group of insects within each dish are “pooled” to get a standard averaged value. This is due to the fact that this averaged value may not be transferrable to insecticides with different modes of action. In our tests the high knockdown for cyfluthrin was expected, and the average value of 2.2 indicates a “heavy” knockdown. Conversely, in the case of chlorfenapyr, where there is no knockdown, the value of 2 consists of the average of the numbers of individuals that were “dead” (1), and normal (5). But, since there was little knockdown, this value may not be relevant. Hence, depending on the insecticide, the index may over- or underestimate knockdown, and, as a result, may not be always a reliable indicator of the use of knockdown as a predictor or mortality. We suggest that these dissimilarities among insecticides can be reflected more accurately by the deviation of values from the average rather than the average value itself. Arthur^21^ found that knockdown was reduced with increasing post-application period, which is probably mainly due to insecticidal dissipation. Also, Arthur^22^ found that deltamethrin was more toxic to *T. castaneum* than to *T. confusum*, which was noted here for some of the combinations tested. Nevertheless, both species are able to recover with ease after knockdown^13,21,22^. Although knockdown has received criticism as it stops the contact of insect with the toxic agent, which may lead to recovery^3^, the absence of knockdown may pose more risks. In an earlier study, Guedes et al.^17^ tested chlorfenapyr against stored-product psocids and reported that there was a delay in the movement, as compared with the untreated controls. This delay may eventually mean that the “critical” exposure of chlorfenapyr may be rather short.

We found that photoperiod did not affect much mortality, under the experimental conditions tested here. Previous studies did indicate that photoperiod may play a critical role in some insecticides that are applied on surfaces. Vassilakos and Athanassiou^23^ found that the efficacy of bacterial insecticide spinetoram on surfaces for the control of *T. confusum*, *S. oryzae* and the saw-toothed grain beetle, *Oryzaephilus surinamensis* (L.) (Coleoptera: Silvanidae) was reduced when these surfaces were exposed to light, as compared with non-illuminated surfaces. Moreover, in that study, the authors reported that this reduction was generally gradual with the increase of the post-application time, while spinetoram was less effective on concrete in comparison with steel^23^. Similar results have been reported for a commercial formulation of cyphenothrin and prallethrin by Karanika et al.^24^ for the control of the above three species, and *P. truncatus*. However, the dissipation of an insecticide on a given substrate does not always relate to reduced efficacy. For spinetoram, Vassilakos et al.^25^ found a reduction in residues of spineroam on grain occurred with post-application periods, but this reduction was not directly correlated with a reduction in efficacy. The results of our study indicated no loss of efficacy of either insecticide during the 5-week post exposure period. This could be attributed to the fact that the entire experimental period on which the dishes remained at the different illumination conditions was rather short, and/or that the two insecticides tested here were rather stable for this period of time.

We demonstrated different levels of knockdown and that knockdown, even at its highest level did not necessarily lead to an increase in mortality, and may not always serve as a reliable indicator of mortality. Mortality can be quantified and is objective, while knockdown assessments are still subjective and cannot be quantified. Although the indicators that are based on knockdown are observer-based, there are good paradigms that these methods are reliable, and provide indices that can be used with success to predict insecticidal efficacy and insect mortality levels^5–7^. More recently, Agrafioti et al.^26^ found that visual observations regarding behavioral changes in stored-products beetles after exposure to phosphine, could be correlated well with automatic behavioral observations that were taken through the Ethovision software. In the context of our study, though knockdown was assessed quantitatively through the development of an index, other factors apparently exert more of an effect on eventual mortality than solely looking at knockdown.

## Materials and methods

### Insects and insecticides

The *T. castaneum* and *T confusum* adults used in the study were obtained from stock cultures reared at the USDA-ARS Center for Grain and Animal Health Research (CGAHR), Manhattan, KS, USA, on a diet of 95% whole wheat flour and 5% Brewer’s yeast. Both species had been in culture at the CGAHR for about 30 years. The two species were reared in continuous darkness inside a chamber (Percival, Perry, IA, USA) set at 27 °C and 60% relative humidity (RH). Only 1–2-week-old mixed sex adults were used for the study. The insecticides were cyfluthrin (Tempo SC Ultra, Bayer Crop Science, USA, 11.8% of active ingredient [AI], 120 mg AI/ml) and chlorfenapyr (Phantom EC, BASF Corporation, Research Triangle Park, NC, USA, 21.45% of active ingredient, 240 mg AI/ml).

### Treatment factors

The bottom portion of plastic Petri dishes (62 cm^2^ in area, 15 mm high) were used as the experimental arenas for the tests. These exposure arenas were created using a driveway patching material (Rockite, Hartline Products, Cleveland, OH, USA) as the surface substrate. Water was mixed with the concrete to create a liquid slurry, and was poured into an individual arena to a depth of about 1.25 cm. The sides of each arena were lined with fluon (Sigma-Aldrich co.) to minimize escape of insects. A total of 120 arenas were created for the tests.

The insecticides were formulated as follows. There are two label rates for cyfluthrin EC, a high rate of 16 ml in 3.8 L to cover 94 m^2^ (20 mg AI/m^2^), and a low rate of 8 ml in 3.8 L to cover 94 m^2^ (10 mg AI/m^2^). This is an equivalent volume rate of about 0.3 ml of formulated insecticide per the surface area of the concrete arena. The amount of formulation needed for 0.3 ml is too small to accurately measure, so for the high rate 0.1 ml of formulation was mixed in 25 ml water, and for the low rate 0.1 ml was mixed with 50 ml of water.

Label directions for formulating chlorfenapyr were to create a 0.5% diluted concentration by mixing 89 ml of product in 3.8 L of water to use as the maximum rate for outside peripheral treatment. This dilution is to be applied at a volume rate of 35.4 ml per 1858 cm^2^. Hence, the equivalent volume spray for the 62-cm^2^ area of the concrete arena was 1.2 ml, which was 6.8 mg AI per Petri dish or 1.1 g AI/m^2^. For our study, the 0.5% dilution was formulated by mixing 1.12 ml of chlorfenapyr in a 50-ml volumetric flask, in proportion to the label directions for mixing the larger volume of 3.8 L. Applying this solution to the arenas at the same volume rate of 0.3 ml per arena as was used for cyfluthrin results in a further 75% reduction in AI to 110 mg AI/m^2^. A solution of half of this rate was created by mixing 0.61 ml of the formulation in 50 ml water. Thus, there were two rates for each insecticide. A final treatment of untreated controls was to be treated with distilled water at the same volume rate of 0.3 ml per arena (5 treatments, insecticides and control).

One of the objectives of the study was to evaluate light intensity as a factor in residual insecticide efficacy. Three Percival incubators (Perry, IA, USA) were prepared at 27 °C, 60% RH with three light:dark cycles, 0:24, 8:16 or 16:8. There were four fluorescent bulbs (Alto 700 series, F32T8/TL741, 32 W, Philips, USA) per incubator, providing light intensity (illumination), as measured in the center of the incubators, at 2400 lx. For an individual replicate, there were 5 insecticides, 2 species, 3 light:dark cycles, and 6 arenas for each treatment, i.e. 180 arenas per replicate. The arenas were treated using artist’s air brushes (Badger corporation, Franklin Park, IL) to mist the solutions onto the concrete surface. The untreated controls were treated with 0.3 ml of distilled water. Then, a separate airbrush was used to treat 6 arenas with the low rate of cyfluthrin, then the airbrush was rinsed with water and 6 arenas were treated with the high rate of cyfluthrin. The airbrush was then thoroughly rinsed with isopropyl alcohol, then the application process was repeated for chlorfenapyr. Then, the entire process was repeated four more times, creating separate insecticide solutions each time, for a total of 4 replicates, i.e. 720 arenas total. All arenas were allowed to dry overnight on a laboratory counter at room temperature of about 25 °C, in continuous darkness.

### Insect exposures

The next day, ten 1–2-week old mixed-sex of *T. castaneum* were added to each of 15 arenas for each replicate, and knockdown was assessed after 5, 15, 30, 60, and 120 min of exposure according, through classifying the exposed adults as knocked down or normally moving.

The exposure times were “staggered” for each replicate to minimize overlap between exposure times. After the exposures were done for *T. castaneum*, the process was repeated for the exposures of *T. confusum*. At the conclusion of the final exposures of *T. confusum*, the replicates were divided equally into separate lots of 40 arenas, and each set placed in one of the three incubators for the different light:dark cycles. After 1 day arenas were removed from the incubators, and knockdown was assessed, according to the following index:

1. No movement.
2. Minimal movement of the antennae and tarsi and very limited body movement,
3. Vigorous movement of the legs and body but the adult could not stand upright.
4. The adult could move and stand upright but could not move more than a few steps before falling over.
5. The adult could move normally, similar to untreated controls.

After the termination of this procedure, all arenas were returned to the incubators. The arenas were assessed again after 3 days and returned to the incubators. After 7 days, a final assessment was made, and the adults were discarded. A new set of adults was put on the arenas, and this process of assessing knockdown at 1, 3 and 7 days was repeated for 5 successive weeks.

### Data analysis

Data were analyzed using IBM SPSS 26.0 (IBM Corp., Armonk, NY, USA). The General Linear Model (GLM) was used for the assessment of the effects of insecticide, rate, light, exposure, time (days) and species to the 1–5 score by considering that the scores represent values that correspond to an underlying continuum. This is a standard practice in studies based on a Likert-scale type assessment^27^. In a similar manner, GLM was used for the assessment of the influence of insecticide, rate, light, exposure and time (weeks/days) to the number of the knocked down adults for both species. Poisson regression was also used for the evaluation of the influence of the different factors to the number of knockdown counts. Equivalent results were obtained; however, we report GLM data since assumptions in model diagnostics were better met. Moreover, Likert-scale data for the adults of both species in the control dishes (without insecticide) are not reported, given that in the vast majority of the cases, all adults in those dishes moved normally, and classified as “5”.

### Ethical approval

This article does not contain any studies with human participants performed by any of the authors. All applicable international, national, and/or institutional guidelines for the care and use of animals were followed.
